# The interaction of regulated forms of cell death in the pathogenesis of severe facial paralysis and potential therapeutic strategies

**DOI:** 10.3389/fnins.2025.1720961

**Published:** 2025-12-12

**Authors:** Xing Tang, Ziqin Ji, Xueling Xiao, Tongtong Zhu, Shoujen Lan, Yeayin Yen, Xin Shao, Shune Tan, Chao Wang, Jicheng Zhang

**Affiliations:** 1 College of Acupuncture-Moxibustion and Tuina, Chengdu University of Traditional Chinese Medicine, Chengdu, China; 2 Medical College, Jiangsu University, Zhenjiang, Jiangsu, China; 3 Sub-Health Clinical Medicine Research Center, Sichuan Integrative Medicine Hospital, Chengdu, China; 4 Department of Healthcare Administration, Asia University, Taiwan, China; 5 Department of Acupuncture, Sichuan Integrative Medicine Hospital, Chengdu, China

**Keywords:** facial paralysis, regulated cell death, neuronal degeneration, neuroprotective effect, targeted therapy

## Abstract

Neuronal cell death plays a central role in the pathogenesis of facial paralysis. In the constructed severe facial paralysis model, axonal damage becomes the key factor triggering retrograde neuronal degeneration, resulting in a large number of neuronal deaths, which seriously affects the function of the facial nerve. The basic fibroblast growth factor exhibits strong neuroprotective ability and can significantly reduce the neuronal mortality rate, providing a strong guarantee for neuronal survival. Viral infection is also an important pathogenic factor that cannot be ignored. Viruses such as herpes simplex virus type 1 can trigger neuroinflammation through the immune response, further exacerbating nerve damage. However, recent studies have also brought hope. Neural reconstruction techniques, targeted drugs, and stem cell therapies hold potential value in promoting the recovery of damaged neural functions. These research results reveal that multiple factors affect the survival and function of neurons in facial paralysis through different pathways, laying a theoretical foundation for targeted treatment against neuronal death. In the future, based on these mechanisms, developing new therapies will bring new treatment opportunities for patients with severe facial paralysis, potentially improving their prognosis and significantly enhancing their quality of life, with important clinical value.

## 1 Introduction

Facial paralysis is a disease that significantly affects the quality of life and facial functions of patients (Iwata et al., 2025). It has attracted much attention due to its typical symptom of facial muscle paralysis. Neuronal cell death is an important factor in the pathological process of facial paralysis, which leads to damage to the facial nerve function. However, facial paralysis is not solely caused by cell death. Ischemic, compressive, metabolic, autoimmune, infectious, and iatrogenic mechanisms may also lead to severe facial paralysis (Wang et al., 2025). For instance, various mediators released during inflammatory responses can attack nerve cells. Viral infections may directly invade the nerve and damage its structure. Ischemia deprives the facial nerve tissue of adequate nutrient and oxygen supply. Metabolic disorders interfere with the normal metabolic process of the nerve. Compression factors impede the normal conduction of the nerve, and iatrogenic factors such as improper surgical or injection operations can also damage the nerve (Table 1). When severe nerve damage occurs, such as axonal rupture, or viral infection, like herpes simplex virus type 1 (HSV-1), facial nerve cells are easily damaged and die, thereby causing facial muscle movement dysfunction and triggering facial paralysis. Clinically, stratifying the severity of severe facial paralysis is of great significance. The House-Brackmann grading system provides precise quantitative standards for clinical condition assessment. Through this stratified description, doctors can more comprehensively and accurately grasp the patient’s condition, formulate personalized treatment plans, making the research closely align with clinical reality and effectively enhance the clinical guiding value (Table 2). Traditional treatments mainly focused on alleviating symptoms, such as using drugs to reduce facial pain, swelling, and other discomforts. Corticosteroids play a crucial role in the treatment of acute peripheral facial palsy. It is currently the only drug therapy that has been proven to have clinical efficacy. From a mechanism perspective, corticosteroids have a close interaction with the regulated cell death (RCD) pathway. They can inhibit major pro-inflammatory cytokines, such as IL-1β, IL-6, and TNF-α, reduce neuronal edema, and stabilize the blood-neural barrier. This series of effects can effectively alleviate immune-mediated neuronal apoptosis and necrotic signal transduction. Early use of corticosteroid therapy can limit inflammation-induced RCD and protect the survival ability of neurons.

**TABLE 1:** Main clinical types of severe facial paralysis.

Classification	Type	Cause of the disease	Key pathogenic mechanisms	Typical clinical manifestations:	Potential therapeutic strategies
Peripheral facial paralysis	Traumatic type	Traumatic factors such as axonal rupture	Axonal damage triggers retrograde degeneration of nerve cells	Complete paralysis of facial muscles, inability to perform actions such as frowning, closing eyes, puffing cheeks, or whistling, with a slow and difficult recovery process, and possible permanent facial muscle dysfunction may remain	Neural reconstruction techniques (such as nerve transplantation, nerve anastomosis), application of growth factors (to promote nerve regeneration)
	Viral infectivity	Infections such as Herpes Simplex Virus Type 1 (HSV-1)	Viral infection activates the immune response, leading to neuroinflammation	Severe facial pain, obvious local swelling, accompanied by facial muscle paralysis. Initially, it may be partial paralysis, and as the condition progresses, it can develop into complete paralysis. Other symptoms such as ear pain and hearing loss may also occur	Antiviral treatment (such as using antiviral drugs like acyclovir), immunomodulation (such as using glucocorticoids to reduce immune response)
	Exposure to cold or instability of autonomic nerve function	Exposure to cold and unstable autonomic nerve function	Local neurovascular spasm causes ischemia and edema of the facial nerve	Partial or complete paralysis of facial muscles, possibly accompanied by pain behind the ears, with a potentially long recovery process	Thermal therapy, nutritional nerve treatment, acupuncture and other physical therapies
	A few severe and stubborn cases	Intractable cases that do not respond to conventional treatment	Continuous death of nerve cells and difficulty in regeneration	Long-term facial motor dysfunction, significant facial muscle atrophy, stiff facial expressions, inability to express normal facial expressions, severely affecting the patient’s quality of life and social function	Stem cell therapy (such as mesenchymal stem cell transplantation, promoting nerve repair and regeneration), targeted drugs (treating based on specific molecular targets)
Central facial paralysis	Related to cerebrovascular diseases	Stroke, cerebral hemorrhage, etc.	Damage to the central nervous system leads to a large number of deaths of facial nerve cells.	Facial muscle weakness is usually manifested in the contralateral facial muscles, accompanied by symptoms such as deviation of the mouth corner and drooling. At the same time, it may also be accompanied by other neurological deficits, such as limb weakness, sensory impairment, and slurred speech. In severe cases, it can lead to coma.	Cerebrovascular disease treatment (such as thrombolysis, thrombectomy, controlling blood pressure and blood sugar, etc.), neuroprotection (using neuroprotective agents to reduce nerve cell death)
	Related to intracranial tumors	Compression or infiltration by intracranial tumors	Tumor compression or infiltration causes ischemic and hypoxic death of nerve cells.	Facial muscle paralysis is often progressive in nature and may be accompanied by symptoms of increased intracranial pressure, such as headache, vomiting, and optic disk edema. In severe cases, it may also present with symptoms of consciousness disturbance and epileptic seizures.	Tumor resection (resecting the tumor completely to relieve pressure on the nerves), nerve repair (such as nerve transplantation, nerve release, etc.)

**TABLE 2:** Stratification of the severity of severe facial paralysis.

Classification	Grade name	Degree of functional impairment	Static manifestations	Dynamic manifestations	Clinical indication
Level IV	Moderate to severe functional impairment	The facial motor function is severely damaged, but some observable movement signs can still be observed.	Static asymmetry, such as drooping of the corners of the mouth, with the overall appearance of the face showing a significant asymmetrical state	Significant limitation in dynamic movement, inability to complete eye-closing action completely, and associated with accessory movements (i.e., while performing a certain facial movement, involuntary triggering of other unrelated facial muscle movements) or facial muscle spasms (uncontrolled, paroxysmal twitching of facial muscles)	Indicates that the condition is quite serious, with significant nerve damage. Active and effective treatment measures, such as drug therapy, physical therapy, and rehabilitation training, need to be taken to improve facial function as much as possible and prevent further deterioration of the condition.
Level V	Severe functional impairment	The facial motor function is extremely limited, with only extremely weak movement capabilities remaining.	Static obvious asymmetry, with the facial asymmetry being more severe than grade IV, which may affect the patient’s appearance and bring them significant psychological pressure	Only slight facial movements remain, such as slight twitching of the corners of the mouth, and eye-closing action cannot be completed at all	Indicates that the nerve damage is severe and recovery is difficult. The treatment should comprehensively consider various methods, such as nerve repair surgery and neurotrophic therapy. At the same time, enhanced rehabilitation care is necessary to prevent complications.
Level VI	Complete paralysis	The face has completely lost the ability for autonomous movement, and the muscles are in a state of complete paralysis.	The face shows no autonomous movement, presenting a completely symmetrical “mask-like face” appearance with no expression changes	No observable facial movements at all, muscles are completely paralyzed, and no facial actions such as eye closure, puffing cheeks, or blowing a whistle can be performed	The condition has reached its most severe stage. The nerve damage may be irreversible. The treatment focus is on improving symptoms as much as possible, preventing complications, and conducting long-term rehabilitation training to enhance the patient’s self-care ability. At the same time, attention should be paid to the patient’s mental health and providing psychological support.

With the continuous deepening of research on facial paralysis, treatment strategies have also undergone significant changes (Noda et al., 2024). In recent years, the focus of treatment has gradually shifted toward neuroprotection and regeneration, and targeted intervention has become a new research hotspot. New treatment methods such as growth factor application, immune regulation, and neural reconstruction technology have continuously emerged, bringing new hope to the treatment of facial paralysis (Huppenbauer et al., 2005). It is worth noting that currently, most of the therapies targeting RCD or promoting regeneration, such as basic fibroblast growth factor, sex hormones, stem cells, platelet-rich plasma, and biomaterials, are still in the preclinical stage and lack data from large-scale human trials to support them. However, these potential therapies have pointed out the direction for future research and are expected to facilitate the transition of more effective medical therapies from the laboratory to clinical practice. At the same time, apoptosis as the primary early driver of neuronal loss, with necroptosis and ferroptosis contributing mainly to inflammation-mediated secondary injury. In-depth exploration of the mechanism of cell death in facial paralysis and understanding of related treatment progress are of great significance for optimizing clinical treatment plans, improving patient prognosis, and guiding the direction of future research. This article reviews the mechanism of cell death in facial paralysis and its treatment progress, aiming to provide theoretical basis for clinical practice and future research.

## 2 The role of RCD in facial nerve injury

Regulated cell death is a key mechanism factor for severe facial paralysis. Among them, apoptosis is the most classic form of RCD, which has a significant correlation in facial nerve injury. When the facial nerve is damaged, various factors can trigger the apoptotic process (Gao et al., 2019). For example, cytokines released at the injury site, imbalance of growth factors, and oxidative stress, can all activate the apoptotic signaling pathways within the cells, such as the mitochondrial pathway and the death receptor pathway. In the mitochondrial pathway, cytochrome C is released into the cytoplasm, activating the caspase cascade reaction, ultimately leading to cell apoptosis. The death receptor pathway directly activates caspase-8 through the binding of cell surface death receptors to corresponding ligands, thereby triggering cell apoptosis. Apoptosis can occur in the early stage of facial nerve injury, resulting in a reduction in the number of neurons and Schwann cells, and affecting the normal function of the nerve. Additionally, necroptosis is also involved. Necroptosis is a form of RCD that combines the characteristics of necrosis and apoptosis. After facial nerve injury, the internal environment of the cells becomes disordered, such as energy metabolism disorders and calcium ion overload, which can induce necroptosis in cells. Unlike apoptosis, necroptosis does not rely on the activation of caspase but forms a necroptotic body through receptor-interacting protein kinases (RIPK) family members, such as RIPK1 and RIPK3, and activates the mixed series protein kinase-like domain (MLKL), causing the cell membrane to rupture and the release of cell contents, triggering an inflammatory response. In facial nerve injury, necroptosis participates in the acute inflammatory response at the injury site, further exacerbating nerve damage.

After facial nerve injury, the activation of the RCD pathway can promote axonal degeneration. During the process of cell apoptosis, apoptotic signals can conduct retrograde along the axon, triggering caspase activation in the local axon, resulting in the degradation of axonal skeleton proteins and axonal rupture. Necroptosis and ferroptosis can also indirectly affect the stability of axons by releasing inflammatory factors and damage signals, accelerating axonal degeneration. Axonal degeneration further aggravates the dysfunction of nerve conduction, leading to the aggravation of facial palsy symptoms. The RCD pathway is also closely related to neural inflammation. During cell apoptosis, apoptotic cells can release “find me” signals, such as phosphatidylserine, to recruit phagocytes to clear apoptotic cells, to some extent, inhibiting the inflammatory response. However, when apoptotic cells are not cleared in time or excessively, apoptotic cells can undergo secondary necrosis, releasing a large amount of DAMPs, activating microglia and astrocytes and other immune cells, triggering neural inflammation. Necroptosis and ferroptosis can directly release DAMPs, activating immune cells, releasing inflammatory factors such as TNF-α, IL-1β, etc., exacerbating the neural inflammatory response. Neural inflammation not only directly damages neurons and Schwann cells, but also forms an inhibitory microenvironment that hinders nerve regeneration. In addition, the continuous activation of the RCD pathway can lead to a reduction in the number of neurons, and the released inflammatory factors and inhibitory molecules can form an environment unfavorable for neuronal regeneration. For example, inflammatory factors can activate glial cells, form glial scars, and hinder the growth and extension of nerve axons. Moreover, the RCD pathway can also affect the proliferation, differentiation, and migration of neural stem cells and progenitor cells, inhibiting neuronal regeneration. The impairment of neuronal regeneration makes it difficult to restore the normal structure and function of the facial nerve after injury, resulting in the persistence of facial palsy symptoms.

## 3 The impact of severe axonal damage on facial nerve cells

The facial nerve, as the key nerve that controls the movement of facial expression muscles, the integrity of its structure and function is crucial for maintaining normal facial expressions and physiological functions. However, when the facial nerve suffers severe axonal damage, a series of complex pathological physiological processes are triggered, among which retrograde degeneration is an important mechanism leading to neuronal cell death (Miller et al., 2012). The axon, as the “bridge” for information transmission between neurons and target cells, once damaged, the distal axon will undergo Wallerian degeneration, while the proximal axon may exhibit retrograde degeneration (Kyriakopoulos et al., 2023). This retrograde degeneration spreads along the axon toward the neuronal cell body, ultimately causing neuronal cell death. After neuronal cell death, not only will the facial muscles it controls lose nerve innervation and present paralysis symptoms, but it will also severely affect the regenerative ability of the axons (Blázquez et al., 2021). Because axon regeneration depends on the normal metabolism and function of the neuronal cell body, after neuronal death, the axon regeneration lacks the necessary substances and energy support, making functional recovery extremely difficult.

In the study of the facial paralysis model induced by freezing, this mechanism was directly verified. The experimental results showed that the death rate of facial nerve cells reached as high as 29%, which fully demonstrated the fatal blow of severe axonal damage to the facial nerve cells (Spiller et al., 2016). However, the intratympanic administration of basic fibroblast growth factor (bFGF) brought new hope for neuroprotection. As an important growth factor, bFGF can promote the survival, growth and differentiation of neurons. In the experiment, after administration of bFGF, the death rate of facial nerve cells significantly decreased to 15.8%, and the facial movement function was also significantly improved (Furukawa et al., 2024). This result suggests that bFGF may exert neuroprotective effects by activating intracellular signaling pathways, promoting the repair and regeneration of neurons, and thereby enhancing the resistance of neurons to damage. Similar research results have also been demonstrated in the facial nerve injury model of hamsters. Sex steroid hormones, such as testosterone and estradiol, exhibit unique effects in neuroprotection (Zaharieva et al., 2016). They can rescue approximately 20% of motor neurons from axon transection-induced cell death through the mechanism mediated by androgen receptors. This indicates that sex steroid hormones have important regulatory roles in the nervous system and may enhance the resistance of neurons to damage by influencing processes such as metabolism and gene expression. The intervention of exogenous growth factors (such as bFGF) and hormones (such as sex steroid hormones) has great potential application value in neuroprotection. Further exploration of their neuroprotective mechanisms will provide new strategies and targets for the treatment of facial paralysis and other nerve injury diseases.

## 4 Synergistic effect of virus infection and immune response

In the pathogenesis of facial paralysis, the synergistic effect of viral infection and immune response plays a crucial role. HSV-1 is one of the common pathogens that cause facial paralysis. The pathogenic process is closely linked to the host’s immune status. Age and immune status are the key factors determining whether HSV-1 infection will lead to facial paralysis (Lee et al., 2025). Taking animal experiments as an example, mice aged 4–5 weeks have an immature immune system and are unable to effectively recognize and eliminate the virus when infected with HSV-1. This results in the virus multiplying abundantly in the body and invading the facial nerve, making it more likely to trigger facial paralysis. In contrast, mice aged 6 weeks have a relatively mature immune system and have high levels of neutralizing antibodies in their bodies (Garcia et al., 2015). These neutralizing antibodies can quickly bind to HSV-1, preventing it from attaching and invading host cells, thus enabling the mice to exhibit resistance to HSV-1 infection and reducing the risk of facial paralysis.

Further research has shown that passive transfer of anti-HSV-1 antibodies or immune T cells can effectively prevent the occurrence of facial paralysis. Anti-HSV-1 antibodies can directly neutralize the virus, while immune T cells can eliminate the virus by recognizing infected cells and killing them. However, this passive immune intervention must be carried out in the early stage of viral infection (Yandong et al., 2024). If viral infection has already triggered a significant immune response and tissue damage, delayed intervention will not be able to effectively prevent facial paralysis. This fully highlights the urgency and importance of early immune intervention.

Apart from HSV-1, other viruses such as Severe Acute Respiratory Syndrome Coronavirus 2 (SARS-CoV-2) and Epstein-Barr Virus (EBV) can also cause facial paralysis. These viruses can not only directly invade the facial nerve, damaging the structure and function of the nerve, but also induce neuroinflammation. During the neuroinflammatory process, T cells, B cells and innate immune cells are activated (Kim and Byrne, 2016). On one hand, these immune cells can exert antiviral effects and eliminate infected cells; on the other hand, excessive immune responses will lead to the massive release of inflammatory factors, further aggravating nerve damage and forming a vicious cycle. It is worth noting that the nerve reconstruction process in severe facial paralysis is influenced by the combined effects of nerve damage, viral infection, and immune response mechanisms (Table 3). If the nerve damage and inflammatory response are too severe, they will impede the normal nerve reconstruction and be detrimental to the recovery of facial paralysis; if the damage and inflammation can be effectively controlled and the anti-inflammatory response is promoted, it may create favorable conditions for nerve reconstruction (Joko et al., 2020). A deeper understanding of this mechanism will help us develop more effective early diagnosis methods and immune intervention strategies, thereby blocking the progression of the disease at an early stage of viral infection and reducing the incidence and severity of facial paralysis.

**TABLE 3:** Molecular biological mechanisms affecting neurological reconstruction in severe facial paralysis.

Mechanism category	The roles of key molecules/cells	Impact
Neural injury mechanism	1. Excessive influx of calcium ions: After nerve injury, the permeability of the cell membrane changes, and a large amount of calcium ions enters the cell, causing an imbalance in the intracellular environment, activating proteases and nucleases, and destroying the cell structure and function.	This leads to damage to the cell structure and function, neuronal death, and triggers severe facial paralysis-related neurological dysfunction.
	2. Retrograde degeneration: Nerve injury triggers retrograde degeneration. The distal axons undergo Wallerian degeneration, and the proximal axons undergo retrograde degeneration, spreading along the axon toward the neuron cell body, ultimately leading to cell death.	
The mechanism of viral infection	Viral infections such as HSV - 1 can enter the neuron cell body through retrograde axonal transport and lie latent in the cell nucleus. When the body’s immunity declines or is stimulated by certain factors, the virus reactivates, releases virus particles, and triggers neuroinflammation and damage.	Triggering neural inflammation, damaging nerve tissues, and exacerbating the symptoms of facial paralysis
Immune response mechanism - Pro-inflammatory response	1. After activation, immune cells release pro-inflammatory factors, mainly including tumor necrosis factor -α (TNF -α), interleukin -1β (IL -1β), interleukin -6 (IL -6), and interferon -γ (IFN -γ). These factors are secreted by activated macrophages, T cells, and neutrophils, and by activating signaling pathways such as NF -κB and MAPK, they induce inflammatory responses.	Excessive inflammatory response directly damages nerve cells, disrupts the blood-brain barrier, allowing more immune cells and inflammatory mediators to enter the central nervous system, forming a vicious cycle that aggravates nerve damage and the severity of facial paralysis.
	2. Chemokines such as monocyte chemoattractant protein -1 (MCP -1) and interleukin -8 (IL -8) can recruit neutrophils and monocytes to the injured site, further exacerbating the inflammatory response.	
	3. Activation of the complement system also generates membrane attack complexes, which directly damage nerve cells and glial cells.	
Immune response mechanism - Anti-inflammatory response	Anti-inflammatory factors include interleukin-4 (IL-4), interleukin-10 (IL-10), transforming growth factor-β (TGF-β), etc. These factors exert neuroprotective effects by inhibiting the production of pro-inflammatory factors or promoting the differentiation of anti-inflammatory phenotype cells.	Exerting neuroprotective effects, reducing neural inflammation and damage, and to a certain extent alleviating the symptoms of facial paralysis

## 5 Neural reconstruction and functional recovery

Facial nerve transection, as a severe type of nerve injury, results in the loss of nerve innervation of facial muscles, causing severe facial paralysis symptoms that significantly affect the quality of life of patients. In this context, neural reconstruction techniques have become a key means for restoring the function of the facial nerve and improving the prognosis of patients (Lyford-Pike et al., 2018). Common surgical procedures include nerve transplantation and facial reconstruction surgery, among others (Table 4). The advantages of nerve transplantation are remarkable. When a long segment of facial nerve is severely damaged due to severe trauma, tumor resection, etc., and cannot be directly anastomosed, it is an extremely effective repair method. By selecting appropriate donor nerves, such as the auricular nerve, sural nerve, etc., and considering factors such as diameter, length, ease of sampling, and impact on the function of the donor area during transplantation, it can reconstruct the nerve conduction pathway and provide the possibility for the recovery of facial muscle function. Relevant clinical research cases have confirmed that it can significantly improve the facial function of patients, allowing them to regain certain facial expressions and movement abilities, and greatly enhancing their confidence and quality of life (Terzis and Anesti, 2011). However, nerve transplantation also has limitations. The surgical operation is complex, and it requires extremely high technical skills from the doctor. Even a slight deviation in key steps such as nerve excision, preparation of the transplantation bed, and nerve anastomosis can affect the surgical outcome. After the surgery, close observation of nerve growth and rehabilitation training are necessary. The recovery period is long and the effect varies from person to person. Some patients may not achieve the desired recovery level. There are various types of facial reconstruction surgeries, each with its own advantages. Muscle transplantation surgery can be used for patients with severe atrophy or loss of facial muscles, to supplement the missing muscle tissue; free tissue flap transplantation surgery can repair large-area facial tissue defects, helping patients restore their facial appearance and some functions, and improving their quality of life. However, these surgeries also have disadvantages. The surgical operation points and techniques are numerous and difficult, and postoperative complications such as infection, hematoma, and necrosis of the transplanted tissue may occur, increasing the patient’s pain and treatment risks. Moreover, different patients have different tolerances and reactions to the surgery, and the surgical effect has certain uncertainty.

**TABLE 4:** Established surgical and reconstruction strategies for severe facial paralysis.

Surgical types	Indications	Surgical methods	Clinical effects
Neural transplantation surgery	Long segment facial nerve defect (such as after trauma or tumor resection), severe facial paralysis that does not respond to conservative treatment	The common donors are the sural nerve and the auricular nerve. Cable-style suturing or inter-neural suture are used. The combined transplantation of the hypoglossal nerve, facial nerve, and cervical 1 nerve can reduce complications.	Restore facial dynamic functions, such as eye closure, blowing cheeks, etc.; combined transplantation of the hypoglossal nerve can reduce side effects like tongue muscle atrophy and improve quality of life
Muscle repositioning surgery	Downward deviation of the mouth corner, facial muscle atrophy	Partial repositioning of the temporal or masseter muscle to repair the corner of the mouth, and re-fixing the muscle attachment points at the corner of the mouth	Immediate visible dynamic effects, but there may be facial asymmetry; suitable for elderly patients or those unwilling to undergo complex surgeries
Static suspension surgery	Severe facial atrophy, cases where nerve-muscle reconstruction is impossible	Using materials such as polytetrafluoroethylene bands and silicone strips to fix between the zygomatic arch and the corner of the mouth, to elevate the drooping tissues	Improve facial contour, but cannot restore dynamic expressions; suitable for patients with high requirements for static functions
Cross-face nerve transplantation surgery	Young patients, requiring autonomous symmetrical expressions	The surgery is divided into two stages: The branch of the healthy facial nerve is ligated with the transfacial transplanted nerve, and then connected to the affected facial nerve after nerve regeneration	Achieve autonomous symmetrical expressions, but multiple surgeries are required; better results for younger patients
Free muscle transplantation surgery	Young patients with high demands for expressions	Transplant the gracilis muscle, latissimus dorsi muscle, etc., with vascular and nerve-rich nerve trunks to the face, and microsurgically ligate the blood vessels and nerves	Restore dynamic functions, but the surgery is complex; there may be muscle fibrosis and long-term rehabilitation training is needed

After nerve injury, the continuous activation of microglia plays a complex and crucial role in the process of neuronal function recovery. Microglia, as immune cells in the central nervous system, are rapidly activated after nerve injury to remove cell debris and secrete neurotrophic factors, promoting nerve repair. However, excessive activation of microglia releases a large amount of inflammatory factors, triggering neuroinflammation and causing further damage to neurons (Rail et al., 2025). Therefore, precisely regulating neuroinflammation and keeping microglia in a moderate activated state may become an important therapeutic target for promoting the recovery of facial nerve function. This suggests that in future research, we need to further explore how to comprehensively apply multiple treatment methods to promote the functional recovery of the facial nerve.

## 6 Application of electrophysiological assessment tools in severe facial paralysis

Severe facial paralysis significantly affects the quality of life of patients. Accurate diagnosis and assessment of the condition are crucial for formulating a reasonable treatment plan (Haginomori, 2023). Electrophysiological assessment tools, such as electromyography (EMG) and neuroelectrography (ENoG), play a key role in the diagnosis of severe facial paralysis, providing important evidence for differentiating reversible from irreversible damage and interpreting the survival status of neurons (Table 5). When axons suffer significant loss due to RCD, the nerve conduction velocity slows down, the wave amplitude decreases, and abnormal spontaneous potentials can be recorded by EMG. The proportion of neurodegeneration shown by ENoG significantly increases, suggesting severe nerve damage and often poor prognosis. Conversely, when the loss of axons is less, the results of electrophysiological examinations are relatively normal, and the prognosis of the patients is usually better. Early inflammation or antiviral treatment plays a crucial role in this process. Inflammation is an important factor driving cell death and can exacerbate the damage of RCD to axons. Early initiation of inflammation or antiviral treatment can effectively inhibit the release of inflammatory factors, alleviate the inflammatory response, thereby inhibiting the RCD driven by inflammation and reducing further loss of axons. As a result, the degree of nerve damage detected by EMG and ENoG will be relatively mild, the possibility of nerve function recovery for the patients will increase, and the prognosis will be more optimistic.

**TABLE 5:** Application of electromyography and neuroelectrography in severe facial paralysis.

Evaluation tools	Basic principle	Normal ranges of each indicator and manifestations of patients with severe facial paralysis	The changes in indicators reflect the condition of the illness.	The significance for clinical decision-making
Electromyography	Record various electrical activities of muscles during rest, voluntary contraction, and when peripheral nerves are stimulated, in order to reflect the bioelectric activities of the neuromuscular system and thereby determine the functional state of the neuromuscular system.	1. At rest: Normal muscles have weak or no resting electrical activity; in reversible damage of severe facial paralysis, it may be basically normal or slightly abnormal, while in irreversible damage, a large amount of self-generated potentials appear. 2. Light contraction: Normal is an interference phase; reversible damage shows a mixed phase or simple phase, the number of motor unit potentials decreases but the recruitment speed is relatively normal; in irreversible damage, the recruitment response is significantly weakened, and the number of motor unit potentials decreases significantly and the recruitment speed is slow. 3. Strong contraction: Normal is an interference phase; reversible damage may still show an interference phase but with reduced amplitude; irreversible damage shows pathological interference phase or simple phase, and the number of recruited motor unit potentials is small and the amplitude is low.	1. The presence of a large number of spontaneous potentials during rest indicates that the muscles have lost innervation, with severe damage and potentially irreversible consequences. 2. The weakened recruitment response during light contractions and the reduction in motor unit potentials suggest a decline in the nerve’s ability to control the muscles, which can assist in determining the reversibility of the injury. 3. The pathological interference phase or simple phase during strong contractions, with few and low-amplitude recruitment potentials, further distinguishes between reversible and irreversible injuries.	1. Distinguish between reversible and irreversible injuries. Take active treatments (such as medication and physical therapy) for reversible injuries to promote the recovery of neural function, and consider surgery or rehabilitation training for irreversible injuries to provide a basis for improving the quality of life. 2. Monitor the treatment effects, promptly adjust the plans, and enhance the scientificity and accuracy of the treatment.
Neuroelectrography	Record the compound muscle action potentials or sensory nerve action potentials generated by nerve trunks under stimulation, and evaluate the nerve function state by measuring parameters such as potential latency, amplitude, and conduction velocity.	1. Latency: Different nerves have different normal ranges. For example, the normal latency of the facial nerve is approximately 3–4 ms; in cases of reversible damage of severe facial paralysis, it may be slightly prolonged, while in irreversible damage, it will be significantly prolonged, and in some cases, no action potential can be elicited. 2. Amplitude: Relatively stable; in cases of reversible damage, it may decrease, but the decrease is relatively small; in cases of irreversible damage, it will significantly decrease, and even disappear. 3. Conduction velocity: Different nerves have different normal conduction velocity ranges. For example, the conduction velocity of the facial nerve is approximately 40–70 m/s; in cases of reversible damage, it may slightly slow down, while in irreversible damage, it will significantly slow down, and even stop conduction.	1. The latency period is significantly prolonged or no action potential can be elicited, indicating severe blockage of nerve conduction, possibly suggesting severe nerve damage or rupture. 2. The amplitude is significantly reduced or disappears, reflecting a decrease in the number of nerve fibers or severe functional impairment. 3. The conduction velocity is significantly slowed down or stops conduction, suggesting severe damage to the myelin sheath or axon.	1. Differentiate reversible and irreversible injuries based on the changes in indicators, and guide the formulation of individualized treatment plans. 2. As an indicator for monitoring treatment effects, adjust the treatment methods according to the recovery status of the indicators to enhance the precision of clinical treatment.

Electromyography is a diagnostic method that records various electrical activities of muscles during rest, voluntary contraction, and when the peripheral nerves are stimulated. It can reflect the bioelectric activities of the nerves and muscles, helping to determine the functional status of the nerves and muscles. Different changes in the displayed electrical potentials can reflect the degree of nerve injury (Schneider et al., 2025). As mentioned above, the appearance of spontaneous potentials, recruitment responses, and changes in motor unit potentials, etc. Generally, the presence of a large number of spontaneous potentials, significantly weakened recruitment responses, a significant reduction in the number of motor unit potentials, and abnormal morphology, indicate more severe nerve injury, possibly irreversible. While relatively mild changes in potentials, with recruitment responses and motor unit potentials changing but still having certain functional preservation, are more likely to be reversible. EMG detects the electrical activities of muscles and indirectly reflects the survival status of neurons. When neurons are damaged but not yet dead, the muscles they control may show certain changes in electrical activity, but still have some functional preservation; while when neurons die, the muscles they control will completely lose nerve control and present typical denervated potential manifestations. Therefore, EMG can infer the survival status of neurons by observing changes in muscle electrical activities, providing important information for clinical treatment.

Neuroelectrography records the compound muscle action potentials or sensory nerve action potentials generated by nerve trunks in response to stimulation. By measuring parameters such as the latency, amplitude, and conduction velocity of these potentials, the functional state of the nerves can be evaluated. The changes in indicators such as nerve conduction velocity, amplitude and latency in ENoG are helpful in differentiating reversible and irreversible injuries. In reversible injuries, these indicators usually change relatively mildly and may recover to some extent after treatment; while in irreversible injuries, the changes are obvious and difficult to recover, such as extremely slow conduction velocity, significant reduction or disappearance of amplitude, and significantly prolonged latency, etc. The indicators of nerve conduction velocity in ENoG are closely related to neuronal function. The normal conduction velocity depends on the integrity of the nerve myelin sheath and the normal function of the axon. When neurons survive but are functionally impaired, there may be mild slowing of conduction velocity and other conditions. While when neurons die or are severely damaged, the conduction velocity will significantly slow down or even stop, and the amplitude will also significantly decrease. Therefore, by detecting the changes in these indicators through ENoG, the survival status and functional state of neurons can be inferred, providing a scientific basis for clinical treatment.

## 7 Other causes and clinical management challenges

In the etiological spectrum of facial paralysis, infectious diseases such as necrotizing otitis externa (NOE) and cranial nerve lesions caused by metastatic tumors are important causes that cannot be ignored, and they pose significant challenges to clinical management. Necrotizing otitis externa, as a severe infectious disease, can lead to facial paralysis (Stanford-Moore et al., 2025). Its prognosis is influenced by multiple factors, among which the control of diabetes is particularly crucial. Due to higher blood sugar levels in diabetic patients, their immune system is weakened, making them prone to infections and making it difficult to control after infection. This results in rapid progression of NOE and an increased risk and severity of facial paralysis (Cho et al., 2022). The type of pathogen is also an important factor affecting prognosis. Fungal infections are more difficult to treat than other pathogens, have longer treatment courses, and may cause more severe damage to the nerves (Ottavi et al., 2025). Moreover, the degree of temporal bone destruction directly relates to the extent and severity of facial nerve involvement. The more severe the temporal bone destruction, the wider the facial nerve damage and the more difficult the functional recovery will be. Therefore, for patients with NOE complicated by facial paralysis, these factors need to be comprehensively considered to develop individualized treatment plans.

Cranial nerve lesions caused by metastatic tumors are often misdiagnosed as Bell’s palsy in clinical practice. Since Bell’s palsy is a common and self-limiting facial nerve paralysis disease, clinicians may easily overlook the possibility of tumor during the initial diagnosis (Lee et al., 2015). However, the cranial nerve lesions caused by metastatic tumors often progress more rapidly and have more severe symptoms. Delayed treatment can lead to further tumor spread, increasing the risk of death for the patients. These cases of facial paralysis caused by special etiologies highlight the necessity of comprehensive differential diagnosis (Bianchi et al., 2010). For patients with refractory facial paralysis, clinicians should conduct detailed medical history inquiries, perform comprehensive physical examinations, and conduct necessary auxiliary tests. For example, through imaging examinations, head enhanced MRI can be used to detect tumor invasion. Through chest, abdominal, and pelvic CT or MRI, the primary tumor lesions can be located. Laboratory tests need to detect tumor markers for auxiliary diagnosis and perform routine tests such as blood routine, liver and kidney function to assess the physical condition. If suspicious lesions are found through imaging, pathological examination should be conducted under permitted conditions to clarify the nature of the lesion. In addition, multidisciplinary collaboration diagnosis is also very important. Inviting doctors from related departments such as neurology, otolaryngology, and oncology for joint consultation is necessary to comprehensively analyze the condition and improve the diagnostic accuracy (Chandan Reddy et al., 2025).

## 8 Potential therapeutic strategies and limitations

Stem cell therapy has shown significant potential in severe facial paralysis. Mesenchymal stem cells (MSCs) and adipose-derived stem cells (ADSCs) are currently the most extensively studied types of stem cells (Oliveira Ferreira et al., 2025). MSCs promote axonal regeneration and myelin repair by secreting neurotrophic factors (such as nerve growth factor NGF) and anti-inflammatory factors. ADSCs enhance axonal regeneration and target muscle reinnervation by differentiating into Schwann cell-like cells. In the rat model, the neural grafts encapsulated with ADSCs significantly increased the number of myelin fibers and the amplitude of electrophysiological activity (Fujii et al., 2020). Moreover, the combined application of stem cells with biomaterials or growth factors can further enhance the therapeutic effect. For instance, the combination of polyethylene glycol (PGA) and ADSCs in the facial nerve transection model resulted in a significantly shorter functional recovery time compared to the group with simple nerve anastomosis. Platelet-rich plasma (PRP) combined with neural-inducing MSCs accelerated axonal regeneration and muscle function recovery by upregulating neurotrophic factors (Cho et al., 2010). Although the results of preclinical studies and early clinical trials are encouraging, the optimization of transplantation strategies, the verification of long-term safety, and the standardization of therapeutic efficacy remain issues that need to be addressed urgently. In the future, through interdisciplinary collaboration, integrating biomaterials, imaging tracking, and personalized medicine, we should promote the transformation of stem cell therapy from the laboratory bench to the hospital bed, ultimately providing more efficient and safe treatment options for patients with facial paralysis.

Similarly, targeted drugs for vestibular schwannomas, such as mTOR inhibitors, have achieved remarkable results in preclinical studies. These drugs can precisely act on specific signaling molecules within tumor cells, inhibiting the growth and proliferation of tumor cells, providing a new and more targeted approach for the treatment of vestibular schwannomas (Chae et al., 2023). However, unfortunately, these drugs have not yet been approved for clinical use. This is mainly because there are still many issues that need to be addressed during the transition from preclinical research to clinical application, such as the long-term safety and efficacy of the drugs in different populations, the determination of the optimal dosage and treatment duration, etc.

Accelerating the transformation of targeted drugs from preclinical research to clinical application is also a key issue that needs to be addressed urgently. Future research can utilize cutting-edge technologies such as gene editing and proteomics to precisely screen drug action targets, optimize drug structure design, and enhance the specificity and efficacy of drugs. During the preclinical research stage, conduct pharmacodynamics, pharmacokinetics, and toxicology studies in strict accordance with international standards to ensure the safety and efficacy of the drugs (Venables et al., 2019). At the same time, actively communicate with regulatory authorities to understand the requirements and procedures for clinical trials and approval in advance, and make preparations for the smooth translation of the drugs. In the clinical research stage, organize multi-center, randomized controlled clinical trials and operate them in strict accordance with GCP guidelines to ensure the authenticity and reliability of the trial data. Through scientific clinical trial design, accelerate the clinical evaluation process of targeted drugs, and provide sufficient evidence for the drug’s market launch.

## 9 Discussion

Facial paralysis is a common disease that significantly affects the quality of life of patients. The underlying mechanism of cell death in this condition is complex and diverse, covering various aspects such as nerve damage, viral infection, immune response, and tumor invasion. It is like a tangled web, presenting great challenges for clinical treatment (Tavares-Brito et al., 2019). A thorough analysis of these mechanisms and the exploration of effective countermeasures are crucial for improving the prognosis and quality of life of patients with facial paralysis. The retrograde degeneration caused by nerve damage is one of the important reasons for the death of facial nerve cells. After axons are damaged, signals are transmitted retrogradely to the cell body of neurons, triggering a series of intracellular events, ultimately leading to the death of neurons, which seriously affects axon regeneration and functional recovery. In terms of viral infection, HSV-1 and others can cause facial paralysis through immune mechanisms, with age and immune status playing key roles (Placheta et al., 2014). Immature mice have a higher susceptibility to the disease, while mice with higher levels of neutralizing antibodies have a certain degree of resistance. Additionally, other viruses such as SARS-CoV-2 and EBV can directly invade the nerves and induce neuroinflammation. The activation of T cells, B cells, and innate immune cells plays a dual role in the progression of the disease (Hattori et al., 2024). Tumor invasion should not be overlooked either; metastatic tumors causing cranial nerve lesions are often misdiagnosed, leading to delayed treatment and increased mortality.

During the process of treating facial paralysis, researchers have made some key discoveries. Growth factors such as basic fibroblast growth factor (bFGF) can reduce the mortality rate of facial nerve cells and improve facial movement function; gonadal steroids can rescue motor neurons through specific mechanisms; immunomodulators such as passively transferred anti-HSV-1 antibodies or immune T cells can prevent facial paralysis, but early intervention is required. End-to-end anastomosis and polyethylene glycol tube anastomosis in nerve reconstruction techniques, as well as polyamine treatment, have to some extent promoted the recovery of facial nerve function. However, these treatment strategies also have obvious limitations (Volk et al., 2010). For example, most current studies focus on short-term efficacy, lacking a unified and standardized efficacy evaluation system and sufficient long-term follow-up data, which limits the comprehensive evaluation of therapy effects. The clinical translation of stem cell and molecular therapies still faces challenges such as safety, stability of efficacy, and mechanism clarification. The early diagnosis of tumor-related facial paralysis is difficult, which can lead to misdiagnosis and missed diagnosis, and delay the treatment opportunity.

Given the current situation and challenges, future research should focus on the development of precise medical strategies. Targeted drug delivery systems can precisely deliver drugs to the affected areas, enhancing drug efficacy and reducing damage to normal tissues (Kim et al., 2023). Individualized immune intervention can be tailored based on the patient’s immune status and disease characteristics, providing personalized treatment plans and improving the specificity and effectiveness of treatment (Rajangam et al., 2024). Through the implementation of these precise medical strategies, it is expected to bring more effective treatment methods to patients with facial paralysis, significantly improving their prognosis and quality of life, and enabling them to return to normal lives. Furthermore, it is necessary to focus on establishing standardized efficacy evaluation criteria, promoting multidisciplinary collaboration, integrating surgical, rehabilitation and biotechnological approaches, in order to achieve the maximum functional recovery and overall improvement of quality of life for patients with severe facial paralysis.
